# Retail liver juices enhance the survivability of *Campylobacter jejuni* and *Campylobacter coli* at low temperatures

**DOI:** 10.1038/s41598-018-35820-7

**Published:** 2019-02-25

**Authors:** Anand B. Karki, Harrington Wells, Mohamed K. Fakhr

**Affiliations:** Department of Biological Science, The University of Tulsa, Tulsa, OK 74104 USA

## Abstract

The high prevalence of *Campylobacter* spp. in retail liver products was previously reported and has been linked to several outbreaks of campylobacteriosis. The main objective of this study was to investigate the influence of retail liver juices on the survivability of several strains of *C. jejuni* and *C. coli*, which were previously isolated from various retail meats at 4 °C. All tested *Campylobacter* strains showed higher survival in beef liver juice (BLJ) and chicken liver juice (CLJ) as compared to beef and chicken juices (BJ and CJ) or Mueller Hinton broth (MHB) at 4 °C. Overall, *C. jejuni* strains showed greater survival in retail liver and meat juices as compared to *C. coli*. CLJ enhanced biofilm formation of most *C. coli* strains and supported growth in favorable conditions. When diluted, retail liver and meat juices enhanced survival of *Campylobacter* strains at low temperatures and increased aerotolerance. In conclusion, beef and chicken liver juices enhanced the survival of *C. jejuni* and *C. coli* strains at low temperatures, which helps explain the high prevalence of *Campylobacter* spp. in retail liver products.

## Introduction

In recent years, campylobacteriosis has been listed as a leading cause of bacterial diarrheal illnesses in the USA^1^. *C. jejuni* and *C. coli* are the primary causal agents for campylobacteriosis, which can lead to immunological disorders like Guillain Barre syndrome and Miller Fisher syndrome^2,3^. After eradication of poliomyelitis, Guillain Barre syndrome remains the leading cause for flaccid paralysis in multiple countries^4–8^. *Campylobacter* is found in various reservoirs and also occurs as a commensal organism in poultry. As a foodborne pathogen, *Campylobacter* is usually transmitted via the consumption of contaminated beverages and food, with the latter occurring primarily from retail meat and liver products^9,10^. Several outbreaks of campylobacteriosis associated with contaminated retail liver products have been reported worldwide^11–17^.

Retail meat and liver products remain important nutrient sources for humans and provide proteins and micronutrients that are essential for growth and immunity^18^. High folate content and various lipid compositions have been identified in beef and chicken liver^19^. The high choline content in retail liver products is beneficial to human health in terms of normal cell functioning and acetylcholine synthesis^20^. In spite of improvements in the handling process, retail meat and liver products may be contaminated with *Campylobacter* during processing in the slaughterhouse^21^. The high incidence of *Campylobacter* on the surface of retail liver suggests cross-contamination during processing; however, it is important to note that *Campylobacter* spp. may also occur in the internal tissues of retail meats^22–24^. Other foodborne pathogens such as *Salmonella, Staphylococcus*, hepatitis E, *Escherichia* spp. and *Yersinia* spp. are also prevalent in retail liver products worldwide^25–29^. The higher prevalence of *Campylobacter* and *Staphylococcus* pathogens in retail liver vs. retail meat products has been reported^25,30^. The high frequency of *Campylobacter* contamination in retail liver products^30–32^ indicates a risk for future outbreaks due to the consumption preference for undercooked liver products^33^. Up to 98% survival of *Campylobacter* has been reported in chicken liver dishes that are undercooked to retain a pinkish coloration^33^.

Although the occurrence of *Campylobacter* in retail meat and liver products largely depends on contamination, bacterial survival during harsh conditions ensures transmission and can result in clinical cases^9,34^. *Campylobacter* is a fastidious, microaerophilic gram-negative bacterium that grows optimally at 42 °C. It routinely encounters various stressors including temperature fluctuation and oxidative and osmotic stress^34^. *Campylobacter* copes with harsh conditions by deploying multiple survival mechanisms including the viable but nonculturable condition (VBNC), biofilm formation and aerotolerance^9,34^. Differential gene expression^35–37^ and the survival of *Campylobacter* in different food matrices^38,39^ indicates the importance of food substances on bacterial viability. Even at low temperatures (0–4 °C), *Campylobacter* can survive for prolonged periods in retail meat and liver^38–41^. Hence, the high prevalence of *C. jejuni* and *C. coli* in retail liver products is associated with enhanced survival at low temperatures. Furthermore, low numbers of *Campylobacter* are sufficient to cause infection^42^. In response to cold shock, genes related to acquisition of cryoprotectants and membrane composition remodeling were abundantly expressed in *Campylobacter*^43^. Various components in retail liver might function as cryoprotectant molecules to improve survival at low temperatures.

Biofilm production is another survival mechanism of *Campylobacter* during aerobic and adverse environmental conditions^44^. The food matrix influences the formation of *Campylobacter* biofilms, which occur in retail meat substances such as chicken and pork meat juices^45,46^. Furthermore, the retail meat environment increases adhesion and attachment of *Campylobacter*, thus facilitating biofilm formation^45^. The nutrient and iron-rich environment in retail liver products might contribute to biofilm formation by *Campylobacter* spp. Although oxidative stress is unfavorable for *Campylobacter* growth, aerotolerance in *Campylobacter* strains would enhance survival in aerobic conditions^47^. Studies on the influence of retail liver environments on aerotolerance and biofilm formation are lacking because the required assays were not readily accessible in food models (e.g. liver slices and homogenates)^48,49^. Liver juices in retail liver samples represent the actual environment that *Campylobacter* strains encounter during handling and storage; however, studies including liver juices as a food model for *Campylobacter* have not been previously undertaken.

In previous studies from our laboratory, a high prevalence of *C. jejuni* and *C. coli* strains were reported in retail meat and liver products^30,32,50^. Interestingly, *C. coli* was recovered from retail beef liver, although beef cuts were not contaminated^30^. Retail liver might contain *Campylobacter* in internal tissues or may get contaminated during processing. In both conditions, higher survival rate of *C. coli* strains in the retail liver environment compared to other food matrices might have contributed to the higher prevalence; however, a precise explanation remains unclear. Most studies on *Campylobacter* survival and gene expression have been conducted with *C. jejuni*^36,37,43,51^ however, it is important to note that genomic differences between *Campylobacter* spp^52^ might be associated with differential survival rates and stress tolerance mechanisms. A few studies have shown higher survival rates of *C. jejuni* vs. *C. coli* strains at a lower temperatures, although contrasting results have been reported^41,48,53,54^. In this study, we investigate the influence of retail meat and liver juices (chicken and beef) on different strains of *C. jejuni* and *C. coli* during biofilm formation, survival or growth at variable temperatures, and oxidative stress (aerotolerance). This study provides further insights regarding the influence of retail meat and liver environments on *Campylobacter* survival.

## Results

### Survival at 4 °C

**Figure 1:**
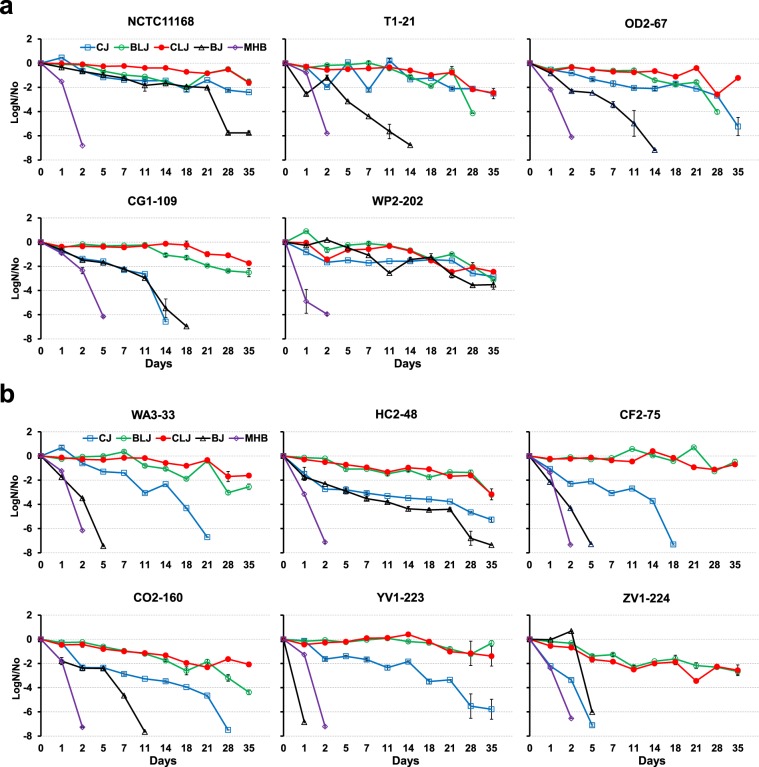
Survival (log CFU/ml) of (**a**) *C. jejuni* and (**b**) *C. coli* strains in beef liver juice (BLJ), chicken liver juice (CLJ), beef juice (BJ), chicken juice (CJ) and MHB at 4 °C. Standard errors (vertical bars) were calculated from mean values of triplicate viable cell counts.

The influence of retail liver and meat juices on the survival of *Campylobacter* strains at 4 °C was investigated. In this experiment, we incubated eleven *Campylobacter* strains including reference strain *C. jejuni* NCTC11168 (Table 1) in retail meat and liver juices at 4 °C for five weeks. Retail meat and liver juices significantly enhanced survival (*P* < 0.0001) in ten of the eleven strains; an exception was *C. coli* YV1-223, which showed higher survival in MHB than BJ. Strains showed higher survival in BLJ and CLJ than BJ, CJ and MHB (*P* < 0.0001) (Fig. 1). Statistical analyses (MANOVA) showed a significant influence of retail meat and liver juices (*P* < 0.0001) but not the origin of juices (chicken vs. beef) on the survival of *Campylobacter* strains. A rapid reduction in bacterial counts (up to 5.76 log reduction) was observed for *Campylobacter* NCTC11168 at 28 days of incubation in BJ (Fig. 1a). A 1.57-, 1.61- and 2.41-log reduction in bacterial numbers was observed for NCTC11168 incubated in BLJ, CLJ, and CJ, respectively, at 35 days of incubation. CJ resulted in higher survival of *C. jejuni* strains T1-21, OD2-67, WP2-202, and NCTC11168 throughout the experiments as compared to MHB and BJ (*P* < 0.0001) (Fig. 1a); however, only *C. coli* strains HC2-48 and YV1-223 survived for 35 days in CJ (Fig. 1b). *C. coli* strains WA3-33, CF2-75, CO2-160, and ZV1-224 did not survive the 35-day incubation period in CJ, BJ or MHB (Fig. 1b). The overall viability of *Campylobacter* strains was lowest in BJ (*P* < 0.0001). None of the strains inoculated in MHB produced visible colonies on Mueller Hinton Agar (MHA) plates after a five-day incubation period.

**Table 1:** *Campylobacter* strains used in this study. All *Campylobacter* strains (except *C. jejuni* NCTC11168) were isolated and whole genome sequenced in our laboratory^30,32,50,78–83^.

*Campylobacter* strains	Source	Accession number (chromosome, plasmids)
*C. jejuni* NCTC11168	Clinical (reference)	Al111168.1
*C. jejuni* T1-21	Chicken meat	CP013116.1, CP013117.1
*C. jejuni* OD2-67	Chicken liver	CP014744.1, CP014745.1, CP014746.1
*C. jejuni* WP2-202	Chicken gizzard	CP014742.1, CP014743.1
*C. jejuni* CG1-109	Beef liver	NA
*C. coli* WA3-33	Chicken liver	CP017873.1, CP017874.1
*C. coli* HC2-48	Beef liver	CP013034.1, CP013035.1
*C. coli* CF2-75	Beef liver	CP013035.1, CP013036.1, CP013037.1
*C. coli* CO2-160	Beef liver	CP013032.1, CP013033.1
*C. coli* YV1-223	Pork meat	NA
*C. coli* ZV1-224	Pork meat	CP017875.1, CP017876.1, CP017877.1

**Figure 2:**
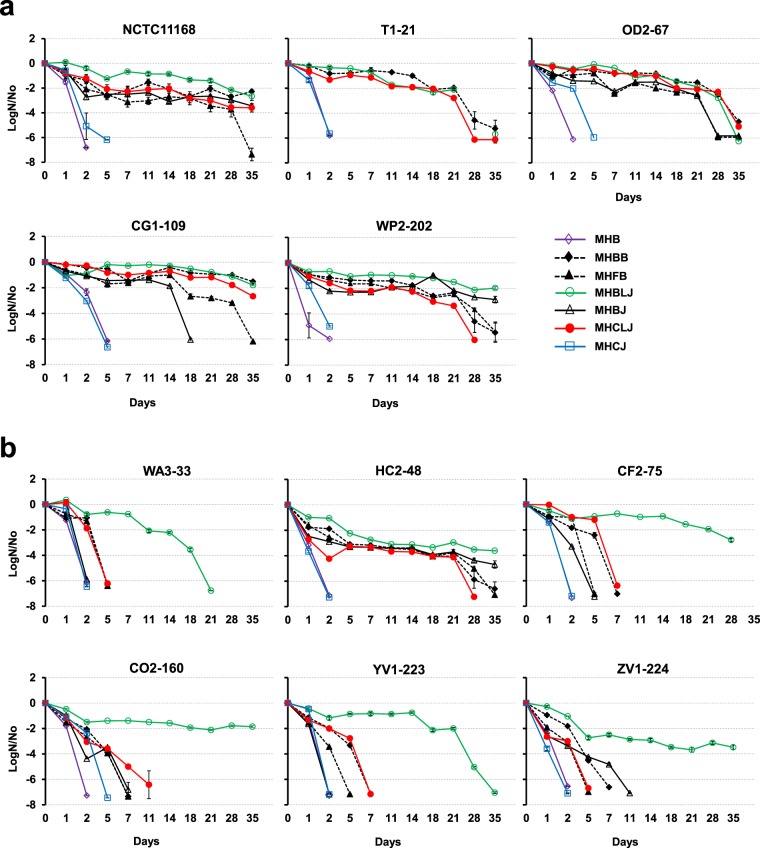
Survival of (**a**) *C. jejuni* and (**b**) *C. coli* strains incubated in diluted retail meat and liver juices (5% v/v in MHB) at 4 °C. *Campylobacter* strains were incubated in diluted beef liver juice (MHBLJ), chicken liver juice (MHCLJ), beef juice (MHBJ), chicken juice (MHCJ), laked horse blood (MHBB), defibrinated fresh horse blood (MHFB) and MHB at 4 °C. Survival curves for *C. jejuni* T1-21 in MHBJ and MHFB are not available. Standard errors (vertical bars) were calculated from mean values of triplicate viable cell counts (LogCFU/ml).

The survival of *Campylobacter* strains in diluted retail meat and liver juices (5% v/v in MHB) was investigated at 4 °C. With the exception of MHCJ, all diluted retail meat and liver juices significantly enhanced *Campylobacter* survival relative to MHB (*P* < 0.0001) (Fig. 2). *Campylobacter* strains failed to grow in MHCJ after 5 days. The addition of 5% laked horse blood and defibrinated horse blood to MHB (MHBB and MHFB, respectively) also enhanced *Campylobacter* survival, with most strains showing higher survival in MHBB vs. MHFB (Fig. 2). For *C. jejuni* strains, survival was highest in MHBLJ, MHBB and MHCLJ than unamended MHB (*P* < 0.0001). Survival rates in MHBB, MHBLJ, and MHCLJ were comparable to 100% BJ for several *C. jejuni* strains (Figs 1a, 2a). Among *C. coli* strains, MHBLJ promoted survival throughout the 35-day experiment. HC2-48 was the only *C. coli* strain producing visible colonies on MHA plates at 11 days after inoculation in MHCLJ, MHBJ, MHBB, and MHFB.

### Survival of *C. jejuni* and *C. coli* in various food matrices at 4 °C

**Figure 3:**
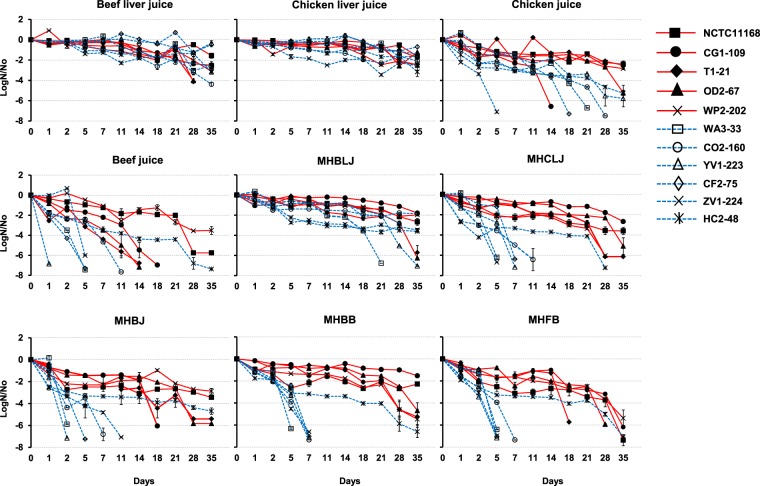
Comparative survival analysis for *C. jejuni* (red lines) and *C. coli* (blue lines) in different retail juices and dilutions at 4 °C. Survival curves represent log reduction (CFU/ml) for each strain at 4 °C (LogN/No). Standard errors (vertical bars) were calculated from mean values of triplicate viable cell counts.

A comparative analysis of viability among *Campylobacter* strains was conducted using log bacterial cell count reduction rates. A higher reduction in bacterial counts (log N/No) was generally observed for *C. coli* strains as compared to *C. jejuni* in variable food matrices. Survival patterns were prolonged for all *Campylobacter* strains incubated in CLJ, BLJ, and MHBLJ (Fig. 3). Interestingly, the survival rate of *C. coli* HC2-48 was significantly higher than other *C. coli* strains in CJ, BJ, MHBJ, MHCLJ, MHBB, and MHFB but comparatively lower than most of the *C. jejuni* strains. No strains survived more than five days in MHB and MHCJ. Two tailed ANOVA for species level differences in individual juices showed significant species level differences of survival rate in retail meat juices (CJ and BJ) and all diluted juices (except MHB and MHCJ) (P < 0.0001). However, MANOVA for interaction including all experimental results for the interaction of *Campylobacter* species, media and time showed significant strains level differences (*P* < 0.0001) but not species-level differences (*P* = 0.38) (see Supplementary Table 1).

### Survival at −20 °C

**Figure 4:**
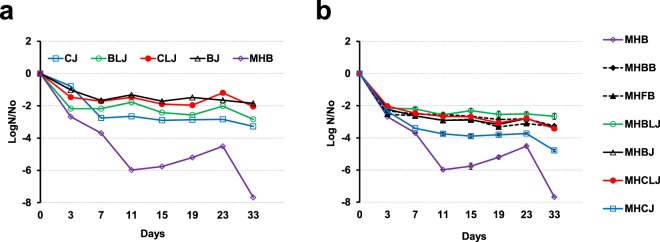
Survival curves for *C. jejuni* NCTC11168 (~7.6 log CFU/ml) inoculated to various full-strength (**a**) and diluted (**b**) retail meat and liver juices (5% dilution in MHB) and incubated at −20 °C. Standard errors were calculated from mean values of triplicate viable cell counts.

Reference strain *C. jejuni* NCTC11168 was used to investigate the influence of retail meat juices on the survival of *Campylobacter* at freezing temperature (−20 °C). We included *C. jejuni* NCTC11168 to compare results with a previous report of survival assay in CJ at freezing temperature (−18 °C)^38^. A rapid decrease in viable cell counts was observed within the first week of incubation (~3.7 logs for MHB, ~2.76 logs for CJ, ~2.17 logs for BLJ, ~1.7 logs for CLJ and ~1.67 logs for BJ); cell counts then remained relatively constant until the end of experiment in most juice matrices (Fig. 4). Diluted retail juices (MHBLJ, MHCLJ, MHBJ, and MHCJ) and horse blood (MHBB, MHFB) increased survival of *C. jejuni* NCTC11168 relative to the unamended MHB media at −20 °C; however, no significant differences were observed in NCTC11168 survival rates among different retail meat and liver juices.

### Growth and survival at 37 °C

**Figure 5:**
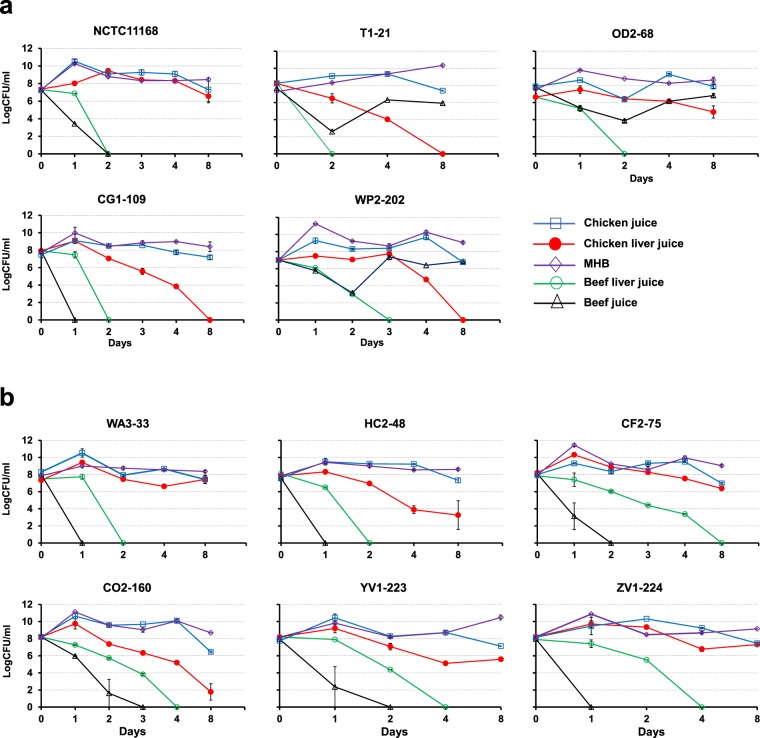
Growth and survival of (**a**) *C. jejuni* and (**b**) *C. coli* strains in full strength (100%) retail juices and MHB at 37 °C in microaerobic condition for eight days. Statistical analyses and standard error bars are based on mean values of log CFU/ml from triplicate experiments.

The influence of retail meat and liver juices on growth and survival of *Campylobacter* strains (Table 1) at favorable growth temperatures was evaluated at 37 °C in microaerobic conditions. As expected, MHB supported higher growth and survival of all strains up to the end of the experiment (Fig. 5). CJ was the best matrix for growth and survival among juices. Chicken liver juice (CLJ) was more favorable for growth at 37 °C than BLJ and BJ for all *C. coli* strains and *C. jejuni* 11168 and CG1-109. None of the strains survived after four days in BLJ. *C. jejuni* NCTC11168 and CG1-109 and all *C. coli* strains failed to survive more than two days in BJ; however, *C. jejuni* T1-21, OD2-67, and WP2-202 survived until the end of the experiment. Interestingly, cell counts of *C. jejuni* T1-21 and WP2-202 decreased at 48 h and then increased, which might be caused by metabolic adaptation to the available nutrients after the initial incubation period. The lower bacterial counts in CLJ, BLJ, and BJ indicated that these matrices were less suitable food sources for growth than CJ in favorable environmental conditions (e.g. 37 °C). MANOVA showed significant species-level differences (*P* = 0.0035). Differences in growth on the basis of strain, juice, and origin of juice were also significant (*P* < 0.0001) (see Supplementary Table 2).

### Influence on biofilm formation

**Figure 6:**
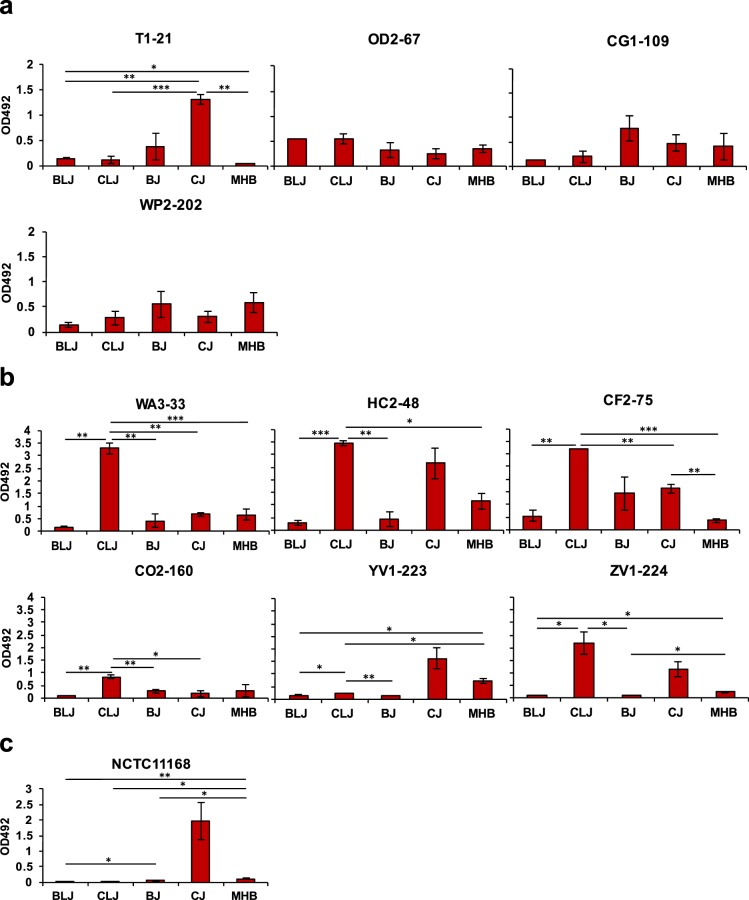
Biofilm formation by (**a**) *C. jejuni* and (**b**) *C. coli*. Strains were incubated at 37 °C in MHB and retail juices (BLJ, CLJ, BJ, CJ) on polystyrene in microaerobic conditions. (**c**) Biofilm assay on borosilicate glass was conducted with *C. jejuni* NCTC11168. Error bars represent the standard error of mean values of absorbance (OD_492_). Significance: *P < 0.05; **P < 0.01; and ***P < 0.001.

On polystyrene surfaces, incubation in CLJ significantly enhanced biofilm formation all *C. coli* strains except YV1-223 (Fig. 6b). Biofilm formation was higher for *C. jejuni* T1-21 and *C. coli* HC2-48, CF2-75, YV1-223 and ZV1-224 in CJ, whereas other strains did not produce significant biofilms in CJ (Fig. 6a,b). All the juices failed to promote biofilm formation in *C. jejuni* OD2-67, CG1-109, and WP2-202. Biofilm formation on borosilicate glass was highest when *C. jejuni* NCTC11168 was incubated in CJ, whereas the other juices did not promote biofilm formation (Fig. 6c). Statistical model analysis showed significant effects of strain-media (*P* < 0.0001) and species-media interactions on biofilm formation (*P* < 0.0001) (see Supplementary Table 3).

### Influence on aerotolerance

**Figure 7:**
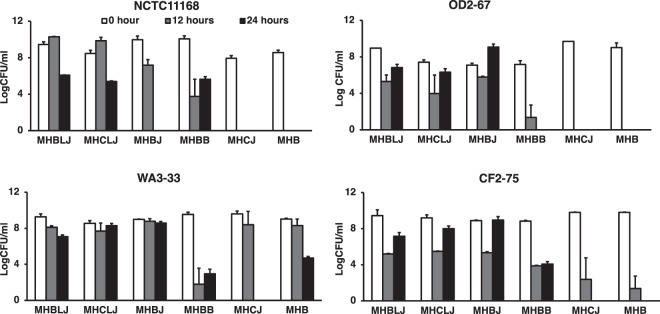
The influence of retail meat and liver juices on aerotolerance of *C. jejuni* NCTC11168 and OD2-67 and *C. coli* WA3-33 and CF2-75. Dilutions (10% v/v) of retail meat and liver juices in MHB were prepared and included MHBLJ (beef liver juice), MHCLJ (chicken liver juice), MHBJ (beef juice), MHCJ (chicken juice). MHBB (10% laked horse blood) and MHB (reference media) were also included in this experiment.

In comparison to non-amended MHB, *C. jejuni* NCTC11168 and OD2-67 and *C. coli* WA3-33 and CF2-75 showed enhanced aerotolerance in MHB amended with 10% beef and chicken liver juice (MHBLJ, MHCLJ) at 24 h of aerobic incubation (Fig. 7). The addition of laked horse blood to MHB (MHBB) enhanced survival in all strains exposed to aerobic conditions but bacterial counts were lower than MHBLJ, MHCLJ and MHBJ; this indicates the presence of an additional factor in retail liver and meat juices that enhances aerotolerance. In general, the addition of BJ to MHB also enhanced aerotolerance, but CJ and non-amended MHB did not.

## Discussion

*Campylobacter* strains routinely encounter low temperatures, and a few studies have speculated that cold tolerance and the acquisition of cryoprotectant molecules are survival mechanisms^43,55^. The prolonged survival of *Campylobacter* in cold storage conditions has been reported in media and food models^38–41,48^, which are influenced by the food matrix composition. Retail meat juices from chicken^38,39,45^, pork^46^ and beef^56^ have been used as models to represent the surface of retail meat products. Analogous to meat juice models, retail liver juice models (BLJ and CLJ) represent the retail liver environment that foodborne pathogens would encounter after contamination. Retail liver possesses choline and other nutrients which are beneficial for human health^18–20^. Heme and nonheme proteins and other nutrients and minerals are also highly abundant in retail meat and liver products^57,58^. The elevated survival of *Campylobacter* in retail liver juices in this study indicates that liver juices provide a nutritious and favorable environment for acquisition of cryoprotectant molecules and nutrients for metabolism^59^ during survival at low temperatures.

During harsh environmental conditions, *Campylobacter* spp. often enter the VBNC state^9^ and undergo a morphological change to coccoid forms^59^. The reported high prevalence in retail liver products^30–32^ and high survival rate at lower temperatures (this study) indicates that the food matrix provided by retail liver products helps retain culturable conditions of *Campylobacter* cells for prolonged periods. Our experimental design represents culturable cell counts of *Campylobacter* on MHA plates after incubation in various media at reduced temperatures. One previous report showed a rise in the *C. jejuni* population in inoculated chicken livers at 4 °C for a 24-h incubation, but a reduction in bacterial numbers was observed in chicken skin medallions and chicken meat^60^. Higher viability (culturability) of *C. coli* strains in retail liver juices vs. retail meat juices (Fig. 1b) correlated with the higher prevalence of reported *C. coli* in retail liver products as compared to retail meat products^30^. Similarly, the high viability of *C. jejuni* in retail liver and meat juices with comparable survival rates (Fig. 1a) might explain the reported high prevalence of *C. jejuni* in retail meats and liver products^30,32,50^. The decline in bacterial counts in our study when using retail liver juices is higher than previous studies, which found no significant reduction in bacterial counts in artificially-inoculated liver samples (liver slices and liver homogenate food models) at 4 °C^48,49,61^. Discrepancies in experimental design (food models used) and differences in the *Campylobacter* strains used in our study might explain the reduced survivability in our study as compared to previous reports.

During the processing and handling of retail meat and liver products, dilutions of substances naturally occur in the working environment and on the surface of retail meat and liver products. In previous studies, media supplemented with blood was compared with full-strength retail meat juices in survival assays^39^ however, the effect of dilutions on survival of *Campylobacter* at lower temperatures has not been previously studied. In this study, 5% dilutions of juices, laked blood and fresh horse blood in MHB were compared to detect the influence of the blood component and additional factor(s) in juices on the survival of *Campylobacter* strains at low temperatures. Enhanced survival of *Campylobacter* in MHB supplemented with 5% laked or fresh horse blood (MHBB or MHFB) (Fig. 2) demonstrates the influence of blood components on survival. The higher survival of selected strains in MHBB vs. MHFB indicates that the lysed blood environment improves survival. The prolonged storage of retail meat and liver products at lower temperatures promotes lysis due to the freeze-thaw process, which creates a more favorable environment for contaminant *Campylobacter* cells. In our study, the higher survival in MHBLJ than MHBB or MHFB for most *Campylobacter* strains (Fig. 2) indicates that BLJ contains other essential nutrients in addition to blood components. For *C. jejuni* strains, all diluents except MHCJ enhanced survival (Fig. 2a); however, only MHBLJ improved the survival of all *C. coli* strains (Fig. 2b). MHBJ enhanced the higher survival of some *C. jejuni* strains more than 100% BJ, which might be due to a more balanced nutritional composition in MHBJ vs. full-strength BJ. None of the *Campylobacter* strains survived well in MHCJ at low temperatures (Fig. 2), which shows the potential loss of the protective or favorable environment observed with full-strength CJ (Fig. 1). These results indicate different nutritional or environmental requirements among *Campylobacter* spp. for survival at low temperatures.

Significantly higher survival of *C. jejuni* than *C. coli* in CJ, BJ, and diluted juices (p < 0.0001) (Fig. 3) supports our contention that the two *Campylobacter* spp. have different nutritional requirements. Our results suggest that *C. coli* might require more nutritional support than *C. jejuni* strains. Hence, highly nutrient rich conditions (like BLJ and CLJ) might have supported similar survival rate for both species which differed for other juices with different nutritional contents. A similar inference was made in a previous report showing the higher survival of *C. jejuni* than *C. coli* in water samples maintained at 4 °C and 20 °C^53^. MANOVA for interaction of species, strains, media and time by including all results for survival at 4 °C, also showed significant strain level differences (*P* < 0.0001). *C. jejuni* strains were previously shown to be more acid tolerant than *C. coli*^62^. Among *C. jejuni* strains, used clinical strains survived better than poultry strains at low temperatures (4 °C and 10 °C) in a previous report^63^. Likewise, a waterborne *C. jejuni* strain showed better survival in defined fresh water media at 4 °C than a foodborne strain^51^. In contrast, a few reports have shown similar survival rates for both species^40,41^ and higher survival rate of *C. coli* at low temperatures in different food models^64^.

At freezing temperatures (−20 °C), all retail liver and meat juices and diluents enhanced survival of *C. jejuni* NCTC11168 relative to MHB (Fig. 4). Hence, retail liver juices likely function as a protective food matrix composition for *Campylobacter* spp. at subzero temperatures. Similar inference for the presence of protective materials in CJ for *Campylobacter* at freezing temperature had been proposed in a previous report^38^. The rapid decrease of bacterial numbers after inoculation into juices maintained at −20 °C (Fig. 4) is similar to previous studies where a rapid decline in bacterial numbers was observed as early as 0.5 h after inoculation^49,54,65^.

At a favorable growth temperature (37 °C), CJ promoted growth at 37 °C for *Campylobacter* strains (Fig. 5a,b), which agrees with previous reports documenting the favorable nutrient composition of CJ for enhanced growth^39^. Chicken liver juice also supported growth and higher survival of all *Campylobacter* strains in comparison to BLJ and BJ (Fig. 5a,b). Although BLJ enhanced survival of *Campylobacter* strains at low temperatures (Figs 1, 3, 4), it did not support growth at 37 °C. Hence, BLJ might provide cryoprotectant molecules and required nutrients for survival at low temperatures, but these factors are not conducive for growth or potentially toxic at favorable temperatures.

To our knowledge, our study is the first to use retail liver juices to investigate the influence of retail liver environments on biofilm formation and aerotolerance, which was not feasible with previously used food models (liver slices and liver homogenates)^48,49,61^. Chicken juice induced high levels of biofilm formation for *C. jejuni* strains NCTC11168 (borosilicate glass surface) and T1-21 (polystyrene) and *C. coli* strains HC2-48, CF2-75, YV1-223 and ZV1-224 (polystyrene) (Fig. 6a–c). In a previous report, full strength as well as 5% dilution of CJ was shown to enhance the attachment of *Campylobacter* strains to abiotic surfaces and biofilm formation^45^. It has been found that CJ environment enhances biofilm formation of both motile and non-motile variants of *Campylobacter*. Among tested juices, remarkably high biofilm formation was seen for most *C. coli* strains in CLJ (Fig. 6b), but *C. jejuni* biofilms were not significantly different in CLJ vs. MHB (Fig. 6a,c). In general, we observed higher biofilm formation among *C. coli* than *C. jejuni* strains in retail juices, which agrees with a previous study^45^. Significant strain-dependent differences in biofilm formation were observed in our study for the various retail liver and meat juices (*P* < 0.0001). Extracellular DNA (eDNA) has been shown to be a major component in biofilm formation of *Campylobacter*, where DNase and eDNase (from *Campylobacter* strains) treatment could rapidly remove or inhibit *Campylobacter* biofilms^66,67^. DNA components available in retail meat and liver juices after lysis of blood, meat and liver cells might enhance biofilm formation of foodborne pathogens like *Campylobacter*. Feng *et al*. previously reported higher biofilm formation of *Campylobacter* spp. in polymicrobial environments than in monomicrobial conditions^68^. Hence, other bacterial contaminants found in retail liver products^25,69^ could contribute to biofilm formation by *Campylobacter* in the retail liver environment.

Although *Campylobacter* spp. are microaerophilic, aerotolerant strains show enhanced survival in aerobic conditions^47^. The oxidative stress response is also associated with the mechanistic basis of iron acquisition^70^. Heme-containing proteins in retail meat and liver products^57,58^ function as cofactors for important enzymes in the oxidative stress response, including catalase and superoxide dismutase^70^. Iron content in media influences the aerotolerance mechanism of *Campylobacter* regulated by regulatory proteins (PerR and Fur) and genes like ferroxoxin (*fdxA)* and ﻿alkyl hydroperoxide reductase (*ahpC)*^71–75^. Hence, the aerotolerance observed for the four *Campylobacter* strains in our study (Fig. 7) might be related to the iron and nutrient content found in diluted (10%) retail meat and liver juices. Although diluted CJ did not enhance aerotolerance; other diluted retail meat and liver juices enhanced survival in aerobic conditions, possibly because of their higher nutrient composition or iron content. It is also important to note that overly high iron levels can promote the formation of toxic superoxide radicals that may be detrimental to *Campylobacter* metabolism^70^. We mention this because it could explain the survival data shown in Fig. 5, where full-strength juices resulted in reduced bacterial counts relative to MHB at 37 °C. The iron content of retail meat and liver juices might also play a significant role in survival at lower temperatures, since the oxidative stress response is activated during cold shock^43^.

Functional metabolic activities and genomic expression data have been reported in *Campylobacter* at lower temperatures (4 or 5 °C) in various growth conditions^35–37^. Similarly, differential expression of genes related to quorum sensing and glycosylation of flagellin have been reported in CJ when compared to artificial Brain heart infusion media^36^. A variety of genes were essential for survival at low temperatures in nutrient-rich or nutrient-poor media^55^. All transcriptomic and genome fit analyses of *Campylobacter* at low temperatures have been conducted with *C. jejuni*^9,36,43,51,55^. Genomic differences between *C. jejuni* and *C. coli* strains^52^ might contribute to differences in survival. Further investigations are needed to validate the effect of genomic differences on survival at low temperatures.

In conclusion, our results show that retail liver juices enhanced the survival of all *Campylobacter* strains at low temperatures, whereas other retail meat juices and dilutions had differential effects on survival. This is a highly relevant finding with respect to food safety since retail liver juices represent an environment encountered by foodborne *Campylobacter* after contamination. Overall, *C. jejuni* strains showed greater survival at 4 °C in chicken juice, beef juice, and diluted retail meat and liver juices as compared to *C. coli*. Chicken liver juice enhanced biofilm formation of most *C. coli* strains and supported growth in favorable growth conditions. Further investigations are needed to explore the mechanisms by which the retail liver environment is enhancing the survival of *Campylobacter* at 4 °C.

## Methodology

### Bacterial strain and growth conditions

*Campylobacter* isolates (four *C. jejuni* and six *C. coli* strains) were used in this study (Table 1) and were previously isolated from retail meat and liver products^30,32,50^. *C. jejuni* NCTC11168 (clinical isolate) was used as a reference strain. The eleven strains were subcultured from −70 °C stock cultures and grown on Mueller Hinton Agar (MHA) supplemented with 5% laked horse blood at 42 °C for 48 h in microaerobic conditions (6% O_2_, 13% CO_2_, 81% N_2_, Thermo Forma incubator, model 3130). Prior to harvesting bacterial cells, strains were further subcultured for 18 h on a fresh plate of MHA with 5% laked horse blood for survival, biofilm and aerotolerance assays. Bacterial cells were harvested in phosphate buffered saline (PBS) (pH 7.4), and cell suspensions were adjusted to OD_600_ = 0.1. In general, bacterial inoculum was prepared similarly for each assay, including survival and growth at variable temperatures, biofilm formation, and aerotolerance.

### Preparation of retail meat and liver juices

For food models, retail meat and liver juices were prepared. Chicken juice (CJ) was prepared as described previously^38,39^. Briefly, frozen retail whole chickens without giblets were purchased from various retail meat shops and thawed overnight at room temperature. A similar procedure was used to obtain beef liver juice (BLJ) and chicken liver juice (CLJ) from frozen beef liver slices and chicken livers, respectively. Beef juice (BJ) was collected from retail meat shops after opening packets containing big chunks of beef cuts. Juices were collected aseptically in sterile containers and stored at −20 °C prior to further processing. After thawing overnight at 4 °C, CJ was centrifuged at 10,000 rpm for 15 min, whereas other meat and liver juices (CLJ, BLJ, and BJ) were centrifuged at 15,000 rpm for 30 min to exclude larger particles. Juices were filter-sterilized with a 0.45 µm membrane filter (Nalgene Rapid-Flow) and stored at −20 °C. The absence of any microbial contaminants in filtered retail meat and liver juices was confirmed by culturing (in aerobic, microaerobic and anaerobic conditions at 25 °C, 37 °C and 42 °C) in MHA supplemented with 5% laked horse blood. Microaerobic and anaerobic incubation at variable temperatures were done in gas jars containing microaerobic gas generating kits and anaerobic gas generating kits (Mitsubishi Gas Chemical, New York, NY, USA) respectively. Dilutions of meat and liver juices (5% or 10% v/v) were also prepared with MHB for survival studies at low temperature and aerotolerance assays. Similarly, dilutions of laked horse blood and fresh horse blood (5%) in MHB were also included to study whether the blood in retail meat and liver products influenced the survival of *Campylobacter* at low temperatures.

### Survival at 4 °C

Appropriate volumes of cell suspensions were added to pre-incubated juices and dilutions to create bacterial concentrations of approximately 7 logs CFU/ml. Strains and log CFU/ml were as follows: *C. jejuni* NCTC11168, 6.63; T1-21, 6.6; OD2-67, 6.98; CG1-109, 6.73; WP2-202, 6.17; and *C. coli* WA3-33, 7.02; HC2-48, 7.28; CF2-75, 7.17; CO2-160, 7.47; YV1-223, 7.14; and ZV1-224, 6.85. MHB was used as a reference medium. An equal volume of inoculated media and juices were filled to the rim of 5 ml disposable polystyrene test tubes with caps and incubated at 4 °C to ensure microaerobic conditions. At specified time intervals, 10 µl samples were taken and serially diluted with 0.1% peptone in saline solution. Two spots of the 10 µl sample from each dilution were spotted onto MHA and incubated at 42 °C for at least 48 h. Viable cell counts were taken, and data analysis was performed with mean values of triplicate experiments.

### Survival at −20 °C

*C. jejuni* NCTC11168 (7.6 log CFU/ml) was used as inoculum in this study for investigating the influence of retail meat and liver juices at freezing temperature. 500 µl of inoculated meat and liver juices (100%), a 5% dilution of meat and liver juices, and MHB were dispensed into 2 ml Eppendorf tubes and maintained at −20 °C. At allocated times, triplicate samples were thawed at room temperature for 10 min and serial dilutions in 0.1% peptone saline were plated. Viable cell counts were taken as described previously.

### Survival at 37 °C

Inoculated juices and MHB (1.5 ml) were dispensed into a 96-well storage plate, (Square Well, 2.2 ml) and incubated at 37 °C in microaerobic conditions. 40 µl samples were removed at specified time intervals and serial dilutions were prepared as described previously. MHA plates spotted with serial dilutions were incubated at 37 °C in microaerobic conditions for at least 48 h before viable cell counts were determined.

### Biofilm formation

Suspensions of all *Campylobacter* strains were prepared and adjusted to OD_600_ = 0.1 in PBS from an 18-h culture. Biofilm assays were prepared as described previously^76^. Briefly, the cell suspension was diluted to 1::10 in meat and liver juice, and MHB was used as a reference media. Biofilm formation on glass surfaces was investigated by incubating 1 ml of *C. jejuni* NTC11168-inoculated media and juices at 37 °C in 10 ml borosilicate glass tubes. After a 72 h-incubation, bacterial cell suspensions were removed, and wells were washed twice with 1.2 ml of sterile PBS (pH 7.4); plates were agitated gently to dislodge unbound cells. MHB (1.2 ml) supplemented with Triphenyl Tetrazolium Chloride (TTC) (0.05% w/v) was added to each well and incubated for 72 h at 37 °C. The other *Campylobacter* strains (excluding *C. jejuni* NCTC11168) were used to evaluate biofilm formation on polystyrene surfaces. Samples (150 µl) were incubated in sterile polystyrene 96-well microtiter plates at 37 °C in microaerobic conditions. After a 72-h incubation, cell suspensions were removed, and wells were washed twice with 180 µl sterile PBS (pH 7.4) and agitated gently to remove unbound cells. MHB (180 µl) supplemented with TTC was added to each well and incubated for 72 h at 37 °C. The remaining MHB/TTC solution was then removed, and wells were air-dried. Bound TTC dye was dissolved using a solution containing acetone (20%) and ethanol (80%); absorbance was measured at 492 nm with an Appliskan Multimode Microplate Reader (Thermo Scientific). All experiments were conducted in triplicate and repeated two or more times.

### Influence of liver and meat juices on aerotolerance

Aerotolerance assays were conducted using four *Campylobacter* strains (*C. jejuni* NCTC11168, *C. jejuni* OD2-67, *C. coli* WA3-33, and *C. coli* CF2-75) as described previously^47^ with minor modifications. Similar approach has been used for aerotolerance assay in previous reports with incubation temperature at 37 °C or 42 °C^71,72,74,77^. Sterilized retail meat and liver juices were mixed with MHB to prepare 10% meat and liver juices. We used 10% dilutions because full-strength (100%) retail meat and liver juices coagulated more quickly, thus hindering assays of viable cell counts. Bacteria were removed from 18-h subcultures on MHA supplemented with 5% laked horse blood. Bacterial suspensions were then diluted to OD_600_ = 0.2 in PBS (pH 7.4). Bacterial suspensions (1 ml) were added to 9 ml of preincubated, diluted juices and then incubated aerobically at 42 °C, with agitation at 200 rpm (New Brunswick I2400 Incubator Shaker). 50 µl samples were removed at 0, 12 and 24 h, and viable cell counts were evaluated on MHA as described previously. All experiments were performed in triplicate.

### Statistical analysis

For survival assays, statistical analysis was conducted with log CFU/ml values of triplicate experiments. Statistical test used with survival data was a repeated measures MANOVA with Strain effect (both *C. jejuni* and *C. coli* strains), Gowth-Media effect, Time effect (cultures repeatedly sampled over time), Strain × Growth-Media interaction, Strain × Time interaction, Growth-Media × Time interaction, and Strain × Growth-Media × Time interaction. The standard methods for approximating the F statistic were used (Wilks’ Lambda test, Pillai’s Trace test, Hotelling–Lawley test and Roy’s Max Root) when needed, which all gave the same statistical conclusion throughout our statistical analysis. For analysis of species level effect on survival, similar statistical test with the survival data was used by a repeated measures MANOVA with species replacing the strain effect (i.e. Species effect, Growth-Media effect, Time effect, Species × Growth-Media interaction, Species × Time interaction, Growth-Media × Time interaction, and Species × Growth-Media × Time interaction).

The initial data analysis was performed with respect to the origin of the *Campylobacter* strains (beef, chicken and pork) and the type of retail liver juice added to the growth media (BLJ, CLJ or none). Repeated measures MANOVA was also done with Origin effect, Juice effect, Time effect, Origin × Juice interaction, Origin × Time interaction, Juice × Time interaction, and Origin × Juice × Time interaction. We then examined whether liver juice specifically was significant [retail liver juice (regardless of origin or none)] by replacing juice origin (beef and chicken) in the analysis. For survival and growth at 37 °C, statistical analysis mirrored that of the survival experiment at 4 °C.

For biofilm assays, the study was conducted with mean values of absorbance (OD_492_). An ANOVA with Strain effect, Growth-Media effect, and Strain × Growth-Media interaction was performed. Student’s t-test and two-tailed ANOVA were conducted for each assay of survival and biofilm as needed.

## Electronic supplementary material

Supplementary Information
