# Supplementary material for: Retail liver juices enhance the survivability of Campylobacter jejuni and Campylobacter coli at low temperatures
Source: Sci Rep. 2019 Feb 25;9:2733. doi: 10.1038/s41598-018-35820-7 (PMC6389972; doi:10.1038/s41598-018-35820-7)
Supplement: Supplementary file 1 — Supplementary Information [file 41598_2018_35820_MOESM1_ESM.docx]

**SUPPLEMENTARY INFORMATION**

**Retail Liver Juices Enhance the Survivability of *Campylobacter jejuni* and *Campylobacter coli* at Low Temperatures**

**Anand B. Karki^1^, Harrington Wells^1^, and Mohamed K. Fakhr^1*^**

^1^Department of Biological Science, The University of Tulsa, Tulsa, OK, 74104, USA

*Corresponding author: mohamed-fakhr@utulsa.edu

**Supplementary Table 1.** Survival Data Analysis.

**1.1.** **Strain:** We first tested for different survival among strains using a Repeated Measures Manova, with repeated culture sampling as the repeated measure. We tested for Strain effect (both *C. coli* and *C. jejuni* strains), Growth-Media effect, Time effect (cultures repeatedly sampled over time), Strain x Growth-Media interaction, Strain x Time interaction, Growth-Media x Time interaction, and Strain x Growth-Media x Time interaction.

**We found significant Strain, Growth-Media and Time effects. In addition all interaction effects were significant.**

**Effect F P**_________

Strain (both *coli & jejuni* strains) F_10,242_ = 143.72 P<0.0001

Growth-Media F_10,242_ = 23.71 P<0.0001

Time (sampled over time) F_10,233_ = 67972.59 P<0.0001

Strain x Growth-Media F_100,242_ = 16.14 P<0.0001

Strain x Time F_100,1680_ = 20.24 P<0.0001*

Growth-Media x Time F_100,1680_ = 22.67 P<0.0001*

Strain x Growth-Media x Time F_1000,2352_ = 11.48 P<0.0001*

* However, while the interaction effects involving Time are technically significant but they are so small compared to the simple Time effect. Hence, we consider them unimportant.

**1.2.** **Species:** Based on the results of the first analysis (above) we went on to test for a species level effect by ignoring strain categories within a species. Again we used a Repeated Measures Manova, with repeated culture sampling as the repeated measure. We tested for Species effect (both *C. coli* and *C. jejuni* strains), Growth-Media effect, Time effect (cultures repeatedly sampled over time), Species x Growth-Media interaction, Species x Time interaction, Growth-Media x Time interaction, and Species x Growth-Media x Time interaction.

**Neither Species nor the interaction Species x Growth-Media were significant. Time and the interactions involving Time were all significant.**

**Effect F P**_________

Species (*coli* vs *jejuni*) F_1,341_ = 0.79 P=0.38

Growth-Media F_10,341_ = 23.45 P<0.0001

Time (sampled over time) F_10,332_ = 22377.04 P<0.0001

Species x Growth-Media F_10,341_ = 0.95 P=0.49

Species x Time F_10,332_ = 5.59 P<0.0001*

Growth-Media x Time F_100,2389_ = 4.98 P<0.0001*

Species x Growth-Media x Time F_100,2389_ = 1.80 P<0.0001*

* However, while the interaction effects involving Time are technically significant but they are so small compared to the simple Time effect. Hence, we consider them unimportant.

**1.3.** **Juice (chicken liver, beef liver or none) and Strain-Origin:** The next stage in our analysis examined whether liver juice or origin of the bacterial strain effected survival. We used a Repeated Measures Manova, with repeated culture sampling as the repeated measure. We tested for Origin effect, Juice effect, Time effect, Origin x Juice interaction, Origin x Time interaction, Juice x Time interaction, and Origin x Juice x Time interaction.

**Neither Origin of the bacterial strain nor the interaction Origin x Time were significant effects. Juice and Times were significant, as were the interactions involving Time.**

**Effect F P**_________

Origin (strain from c, b, p) F_3,351_ = 0.94 P=0.38

Juice (for media from c, b, n) F_2,351_ = 11.95 P<0.0001

Time F_10,342_ = 15693.35 P<0.0001

Origin x Juice F_6,351_ = 0.39 P=0.88

Origin x Time F_30,1005_ = 4.28 P<0.0001*

Juice x Time F_20,684_ = 3.34 P<0.0001*

Origin x Juice x Time F_60,1797_ = 2.11 P<0.0001*

* However, while the interaction effects involving Time are technically significant but they are so small compared to the simple Time effect. Hence, we consider them unimportant.

**1.4.** **Juice (liver juice (regardless of origin) or none) and Strain-Origin:** Finally, we repeated the analysis (1.3 above) but just considered if liver juice without consideration for origin was significant. We used a Repeated Measures Manova, with repeated culture sampling as the repeated measure. We tested for Origin effect, Juice effect, Time effect, Origin x Juice interaction, Origin x Time interaction, Juice x Time interaction, and Origin x Juice x Time interaction.

**This variation did not change the results from the previous analysis. Origin and Origin x Juice were not significant, but the remaining factors and interactions were significant.**

**Effect F P**_________

Origin (strain from c, b, p) F_3,355_ = 0.94 P=0.42

Juice (liver + or -) F_2,355_ = 57.52 P<0.0001

Time F_10,346_ = 18116.81 P<0.0001

Origin x Juice F_3,355_ = 0.40 P=0.75

Origin x Time F_30,1016_ = 8.45 P<0.0001*

Juice x Time F_10,346_ = 14.49 P<0.0001*

Origin x Juice x Time F_30,1016_ = 7.01 P<0.0001*

* Again, while the interaction effects involving Time are technically significant but they are so small compared to the simple Time effect. Hence, we consider them unimportant.

**Supplementary Table 2.** Growth Data Analysis

**2.1.** **Strain:** We first tested for differential growth among strains using a Repeated Measures Manova, with repeated culture sampling as the repeated measure. We tested for Strain effect (both *C. coli* and *C. jejuni* strains), Growth-Media effect, Time effect (cultures repeatedly sampled over time), Strain x Growth-Media interaction, Strain x Time interaction, Growth-Media x Time interaction, and Strain x Growth-Media x Time interaction.

**We found significant Strain, Growth-Media and Time effects. In addition, all of the interactions were significant.**

**Effect F P**_________

Strain (both *coli & jejuni* strains) F_10,110_ = 9.21 P<0.0001

Growth-Media F_4,110_ = 24.01 P<0.0001

Time (sampled over time) F_3,108_ = 15.31 P<0.0001

Strain x Growth-Media F_40,110_ = 7.11 P<0.0001

Strain x Time F_12,286_ = 13.50 P<0.0001

Growth-Media x Time F_30,118_ = 9.63 P<0.0001

Strain x Growth-Media x Time F_120,324_ = 8.56 P<0.0001

**2.2.** **Species:** Based on the results of the first analysis (above) we went on to test for a species level effect by ignoring strain categories within a species. Again we used a Repeated Measures Manova, with repeated culture sampling as the repeated measure. We tested for Species effect (both *C. coli* and *C. jejuni* strains), Growth-Media effect, Time effect (cultures repeatedly sampled over time), Species x Growth-Media interaction, Species x Time interaction, Growth-Media x Time interaction, and Species x Growth-Media x Time interaction.

**Species, Growth-Media and Time were significant effects, as were all of the interactions. This was in contrast to the survival study where Species alone was not a significant effect.**

**Effect F P**_________

Species (*coli* vs *jejuni*) F_1,155_ = 8.78 P=0.0035

Growth-Media F_4,155_ = 10.59 P<0.0001

Time (sampled over time) F_3,153_ = 9.72 P<0.0001

Species x Growth-Media F_4,155_ = 6.03 P=0.0002

Species x Time F_3,153_ = 2.98 P=0.033

Growth-Media x Time F_12,405_ = 4.48 P<0.0001

Species x Growth-Media x Time F_12,405_ = 2.47 P=0.0039

**2.3. Juice (chicken liver, beef liver or none) and Strain-Origin:** The next stage in our analysis examined whether liver juice or origin of the bacterial strain effected culture growth. We used a Repeated Measures Manova, with repeated culture sampling as the repeated measure. We tested for Origin effect, Juice effect, Time effect, Origin x Juice interaction, Origin x Time interaction, Juice x Time interaction, and Origin x Juice x Time interaction.

**Origin, Juice and Time were all significant effects. In addition, all of the interactions were significant. This was in contrast to the survival data where** **the Origin effect and Origin x Juice interaction were not significant.**

**Effect F P**_________

Origin (strain from c, b, p) F_3,153_ = 8.20 P<0.0001

Juice (for media from c, b, n) F_2,153_ = 7.62 P<0.0001

Time F_3,151_ = 16.77 P<0.0001

Origin x Juice F_6,153_ = 6.44 P<0.0001

Origin x Time F_9,368_ = 5.32 P<0.0001

Juice x Time F_6,302_ = 8.70 P<0.0001

Origin x Juice x Time F_18,428_ = 4.30 P<0.0001

**2.4.** **Juice (liver juice (regardless of origin) or none) and Strain-Origin:** Finally, we repeated the analysis (2.3 above) but just considered if liver juice without consideration for origin was significant. We used a Repeated Measures Manova, with repeated culture sampling as the repeated measure. We tested for Origin effect, Juice effect, Time effect, Origin x Juice interaction, Origin x Time interaction, Juice x Time interaction, and Origin x Juice x Time interaction.

**Origin and Juice effects as well as Origin x Juice interaction were not significant. Time and the Origin x Time interaction were significant. Juice x Time and Origin x Juice x Time were not significant.**

**Effect F P**_________

Origin (strain from c, b, p) F_3,157_ = 1.66 P=0.18

Juice (liver + or -) F_1,157_ = 3.25 P=0.073

Time F_3,155_ = 11.72 P<0.0001

Origin x Juice F_3,157_ = 1.87 P=0.14

Origin x Time F_9,377_ = 3.61 P<0.0002

Juice x Time F_3,155_ = 1.15 P=0.33

Origin x Juice x Time F_9,377_ = 1.57 P=0.12

**Supplementary Table 3.** Biofilm Data Analysis

**3.1.** We used an ANOVA with Strain effect, and Growth-Media effect, and Strain x Growth-Media interaction effect on biofilm growth.

**Strain and Growth-Media were both significant effects as was the interaction.**

**Effect F P**_________

Growth-Media F_4,275_ = 124.86 P<0.0001

Strain (*C. coli*) F_10,275_ = 53.15 P<0.0001

Strain x Growth-Media F_40,275_ = 24.57 P<0.0001
